# Increased Presence of FOXP3^+^ Regulatory T Cells in Inflamed Muscle of Patients with Active Juvenile Dermatomyositis Compared to Peripheral Blood

**DOI:** 10.1371/journal.pone.0105353

**Published:** 2014-08-26

**Authors:** Yvonne Vercoulen, Felicitas Bellutti Enders, Jenny Meerding, Maud Plantinga, Elisabeth F. Elst, Hemlata Varsani, Christa van Schieveen, Mette H. Bakker, Mark Klein, Rianne C. Scholman, Wim Spliet, Valeria Ricotti, Hans J. P. M. Koenen, Roel A. de Weger, Lucy R. Wedderburn, Annet van Royen-Kerkhof, Berent J. Prakken

**Affiliations:** 1 Laboratory of Translational Immunology, Pediatric Immunology, Wilhelmina Children’s Hospital, University Medical Center Utrecht, Utrecht, The Netherlands; 2 Division of Allergology, Immunology and Rheumatology, Department of Pediatrics, University Hospital, Lausanne, Switzerland; 3 Rheumatology Unit, University College London Institute of Child Health, on behalf of the Juvenile Dermatomyositis Research Group (UK and Ireland), London, United Kingdom; 4 Department of Pathology, University Medical Center Utrecht, Utrecht, The Netherlands; 5 Dubowitz Neuromuscular Centre, University College London Institute of Child Health, London, United Kingdom; 6 Department Laboratory Medicine, Section Medical Immunology, Radboud University Nijmegen Medical Centre, Nijmegen, The Netherlands; 7 Department of Pediatric Immunology and Rheumatology, Wilhelmina Children’s Hospital, University Medical Center Utrecht, Utrecht, The Netherlands; New York University, United States of America

## Abstract

Juvenile dermatomyositis (JDM) is an immune-mediated inflammatory disease affecting the microvasculature of skin and muscle. CD4^+^CD25^+^FOXP3^+^ regulatory T cells (Tregs) are key regulators of immune homeostasis. A role for Tregs in JDM pathogenesis has not yet been established. Here, we explored Treg presence and function in peripheral blood and muscle of JDM patients. We analyzed number, phenotype and function of Tregs in blood from JDM patients by flow cytometry and *in vitro* suppression assays, in comparison to healthy controls and disease controls (Duchenne’s Muscular Dystrophy). Presence of Tregs in muscle was analyzed by immunohistochemistry. Overall, Treg percentages in peripheral blood of JDM patients were similar compared to both control groups. Muscle biopsies of new onset JDM patients showed increased infiltration of numbers of T cells compared to Duchenne’s muscular dystrophy. Both in JDM and Duchenne’s muscular dystrophy the proportion of FOXP3^+^ T cells in muscles were increased compared to JDM peripheral blood. Interestingly, JDM is not a self-remitting disease, suggesting that the high proportion of Tregs in inflamed muscle do not suppress inflammation. In line with this, peripheral blood Tregs of active JDM patients were less capable of suppressing effector T cell activation *in vitro*, compared to Tregs of JDM in clinical remission. These data show a functional impairment of Tregs in a proportion of patients with active disease, and suggest a regulatory role for Tregs in JDM inflammation.

## Introduction

Juvenile dermatomyositis (JDM) is a rare systemic immune mediated inflammatory disease in which the immune system targets skeletal muscles, skin and sometimes internal organs. The predominant clinical symptoms are muscle weakness and skin rash. JDM has an incidence rate of 3.2 per million in patients aged below 17 in the USA [1] and its cause remains unknown.

JDM is characterized by a highly inflammatory environment proven by the elevation of different chemokines not only in the blood but also in the muscle [2]–[5]. Furthermore, in muscle biopsies of new onset JDM patients activated CD4^+^ T cells are predominantly present, suggesting a pathogenic role for these cells [6]. CD4^+^CD25^+^FOXP3^+^ regulatory T cells (Tregs) are known to be potent regulators of T cell mediated autoimmune responses [7], [8]. For instance, in patients with oligoarticular juvenile idiopathic arthritis (JIA), the presence of Tregs correlates with favorable disease outcome [9]. Little is known about Tregs in classical connective tissue diseases. In studies describing Treg numbers in adults with systemic sclerosis or dermatomyositis, contrasting results have been found [10]–[12] and it remains unclear whether FOXP3 expressing Tregs are present and functional in JDM patients.

It has been shown that Tregs can lose suppressive capacity in an inflammatory environment [13]–[15]. Moreover, under inflammatory conditions human FOXP3^+^ Tregs express pro-inflammatory cytokines such as Interleukin 17 (IL-17) and Interferon γ (IFNγ) [16], [17]. The current treatment of JDM is based on long term general immune suppression, resulting in unwarranted side effects, without curing the disease. As therapies targeting Tregs are currently being developed, it becomes critical to determine the functional role of Tregs in JDM.

To establish whether Tregs play a role in the pathogenesis of JDM, we investigated the presence and function of Tregs in peripheral blood and inflamed muscle of JDM patients.

## Patients and Methods

### Ethics statement

JDM patients were followed up at the UMC Utrecht, the Netherlands, or as part of the JDM Cohort and Biomarker study and Repository (UK and Ireland). DMD muscle biopsies were from the MRC Neuromuscular Biobank. This study was conducted according to the Declaration of Helsinki, and approved by the institutional review boards: Medisch Ethische Toetsingscommissie (METC) UMC Utrecht, and Great Ormond Street Hospital/UCL Institute of Child Health Research Ethics Committee. Written consent was obtained from all participants, and/or their parents (NL 34124.041.10).

### Participants

JDM patients were included according to the Bohan & Peter criteria [18]. 5 patients in the active disease group were diagnosed as JDM patient, but developed in the later follow up a clinical picture of an overlap syndrome (mainly mixed connective tissue disease), 1 of this patient was also included in the remission group. Active disease was defined based on clinical criteria (typical skin manifestations, muscle involvement proved by abnormal Childhood Myositis Assessment Scale (CMAS) score (CMAS<45), and/or an increase in muscle enzymes: Lactate dehydrogenase (LDH), Creatine kinase (CK), Aspartate aminotransferase (AST) [19]. Disease remission was defined for this study as the absence of active inflammation. Healthy children, admitted for minor urological surgical procedures, served as controls. As disease controls patients with Duchennes Muscular Dystrophy were included. Due to evident genetic reasons we could not provide sex matched controls. Patient characteristics are displayed in Table 1.

**Table 1:** Participant characteristics.

		PBMC		Muscle sections		
	JDM remission	JDM active	Controls	JDM active	DMD	
	Fig. 1/Fig. 3	Fig. 1/Fig. 3	Fig. 1	Fig. 2	Fig. 2	
**Number**	27/11	21/11	13	8	9	
**Newly diagnosed**	-	8/4	-	8	9	
**Male (percentage)**	56/73	38/27	69	38	100	
**Median age: years (range)**	11(8–17)/15 (5–17)	9 (5–17)/13 (6–17)	6 (1–13)	6 (1–11)	5 (2–6)	
**Therapy**	22/6	15/9	0	0	0	
Prednisone use	22/5	14/8	-	-	-	
Prednisone low dose (< 0.5 mg/kg/day)	22/5	5/3	-	-	-	
Prednisone high dose (1 mg/kg/day)	0/0	6/2	-	-	-	
High dose corticosteroid pulses (>1 mg/kg)	0/0	3/4	-	-	-	
MTX	13/5	6/1	-	-	-	
IVIG	1/0	1/1	-	-	-	
Other	1/1	2/3	-	-	-	

### Cells and medium

Peripheral blood mononuclear cells (PBMC) were isolated using Ficoll Isopaque density gradient centrifugation, frozen in Fetal Calf Serum (FCS) containing 10% Dimethyl sulfoxide (DMSO), stored up to 1 month at −80°C and following stored in liquid nitrogen for 1 month, up to 8 years. Viability of the samples after thawing was established by trypan blue staining. Samples with >90% viability were included. RPMI 1640 containing 10 mM HEPES, 2 mM L-glutamine,100 U/ml penicillin-streptomycin and 10% human serum was used for culture (Invitrogen, USA).

### Flow cytometry

0.5-1×10^6^ PBMC/ml were stained using the FOXP3 staining kit (eBioscience, USA) according to manufacturer’s protocols with fluorochrome-labeled mAbs against human CD4 (clone RPA-T4), CD25 (clone 2A3), CD127 (clone hIL-7R-m21) IL-17 (clone N49-653), and CTLA-4 (Cytotoxic T-Lymphocyte Antigen 4, clone BN13) (all BD Biosciences, USA), GITR (glucocorticoid-induced TNFR-related protein, clone 110416, R&D, Germany) and FOXP3 (clone PCH101, eBioscience, USA), and analyzed by FACSCanto (BD Biosciences, USA).

IL-17 production was assessed after 3-hour stimulation of cells at 37°C in the presence of phorbol 12-myristate 13-acetate (PMA) (0.05 µg/mL), ionomycin (0.5 µg/mL), and brefeldin A (5 µg/mL) (Sigma-Aldrich, USA).

### Suppression assay

10,000 PBMC were stimulated with plate-bound anti-CD3 (clone OKT-3, 1.5 µg/ml, eBioscience, USA). Autologous CD4^+^CD25^+^CD127^low^ T cells were sorted directly into wells of culture plates by FACS Aria (BD Biosciences, USA) in different co-culture ratios. Cells were co-cultured for 6 days, the last 18 h with [^3^H] thymidine (1 µCi/well). All cultures were performed in triplo or duplo. Suppressive activity was determined as the relative difference in proliferative response (mean [^3^H] counts of triplicate or duplicate wells) compared to PBMC cultured alone. Lower [^3^H] thymidine incorporation in PBMC+PBMC than PBMC alone indicates that the increased number of cells decreases proliferation, leading to exclusion of the assay from the analyses (see Figure S1 and Table S1). Defective suppression is defined as showing no decrease at all or an increase of proliferation in the presence of the sorted Tregs, in at least one of the co-culture ratios.

### Immunohistochemistry

Muscle biopsies were from the upper leg (m. quadriceps or m. vastus lateralis). Stainings were performed on acetone-fixed cryostat-sections with mouse-anti-human CD3 (clone UCHT1, Nova, USA), followed by HRP Rabbit/Mouse (Dako, UK), or with biotinylated rat-anti-human FOXP3, followed by R.T.U.Vectastain kit (Vector Laboratories, UK), and visualized using 3,3'-Diaminobenzidine. Please note that CD3 staining was used to stain T cells, since CD4 expression is not specific to T cells only. 1–4 infiltrated areas were analyzed per patient, depending on the number of infiltrated areas found in the section. Pictures of CD3+ and FOXP3+ stainings were taken of infiltrated areas at 20× magnification, on consecutive slides with the same infiltrated area, and for each picture 2 independent researchers counted all positive cells. The number of cells was adjusted to the number of pictures counted, to obtain an average number per analyzed area for each muscle section.

For immunofluorescent staining secondary antibodies used were: anti-mouse-alexa488, and streptavidin-alexa594 (Invitrogen, USA), pictures were taken at 20× magnification. Stainings of the paraffin embedded muscle sections for IL-17 and FOXP3 analyses were performed according to previously published methods [20].

### Statistical analysis

**Figure 1:**
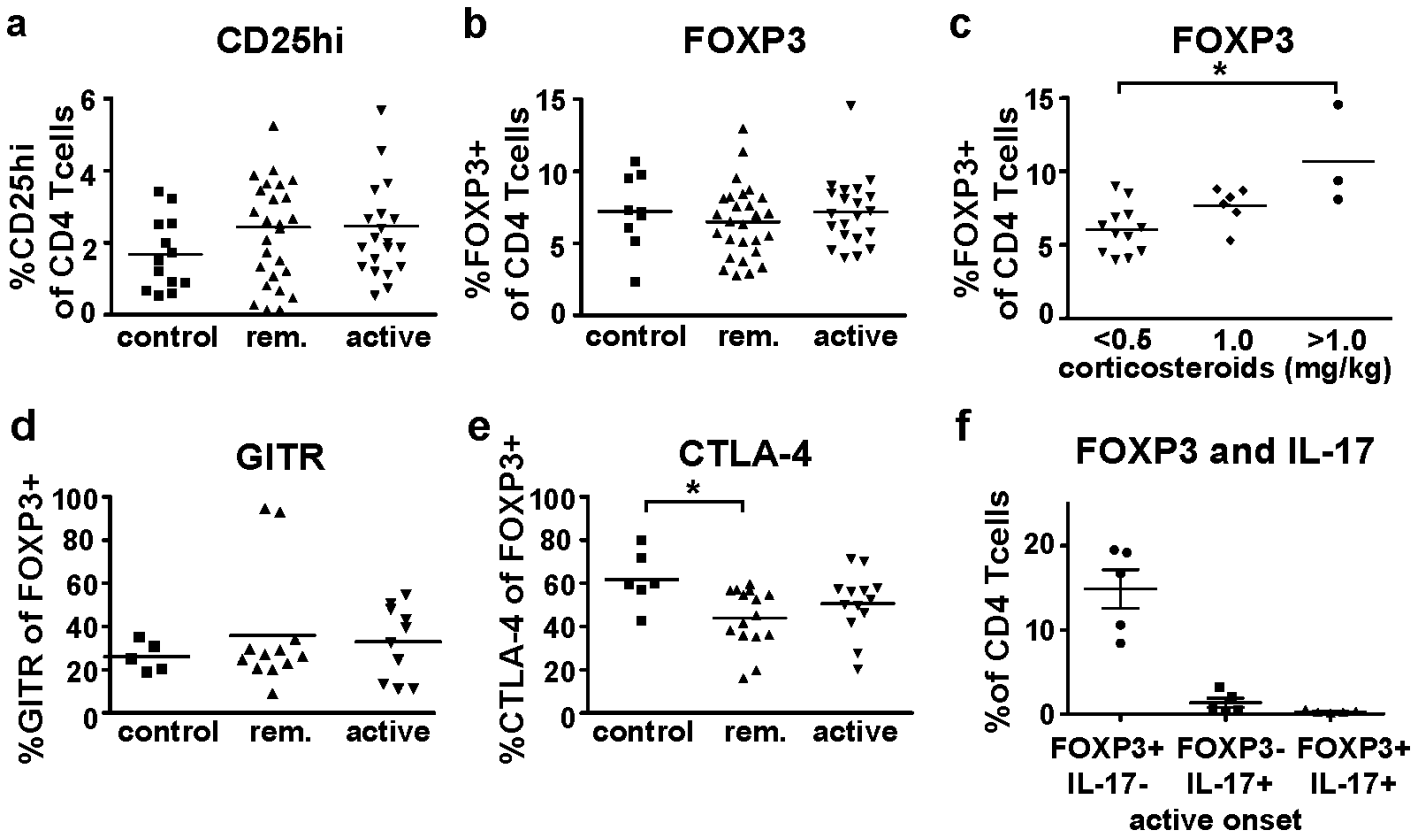
Peripheral blood CD4 T cells from JDM patients and age-matched controls have similar expression of Treg markers. PBMC from JDM patients (JDM) with active (active) or remitting (remission) disease, and controls (control) were isolated and analyzed by flow cytometry (A–F). (A) Percentage of CD25^hi^ (control N = 13, remission N = 27, active N = 21), and (B) FOXP3 (control N = 9, remission N = 27, active N = 21) expressing CD4^+^ T cells. (C) Percentages of FOXP3 expressing CD4^+^ T cells in active JDM patients treated with corticosteroids (<0.5 mg/kg N = 12, 1 mg/kg N = 6, >1 mg/kg N = 3). Percentages of (D) GITR (control N = 5, remission N = 12, active N = 10), and (E) CTLA-4 (control N = 6, remission N = 15, active N = 13) expressing CD4^+^ T cells. (F) PBMC from patients with active JDM (at the time of diagnosis, without medication) were stimulated with PMA and ionomycin and stained for flow cytometry analysis of CD4^+^ T cells expressing FOXP3 and IL-17 (N = 5). All graphs show mean percentages ± SEM. *P<0.05, one way ANOVA analysis was used.

**Figure 2:**
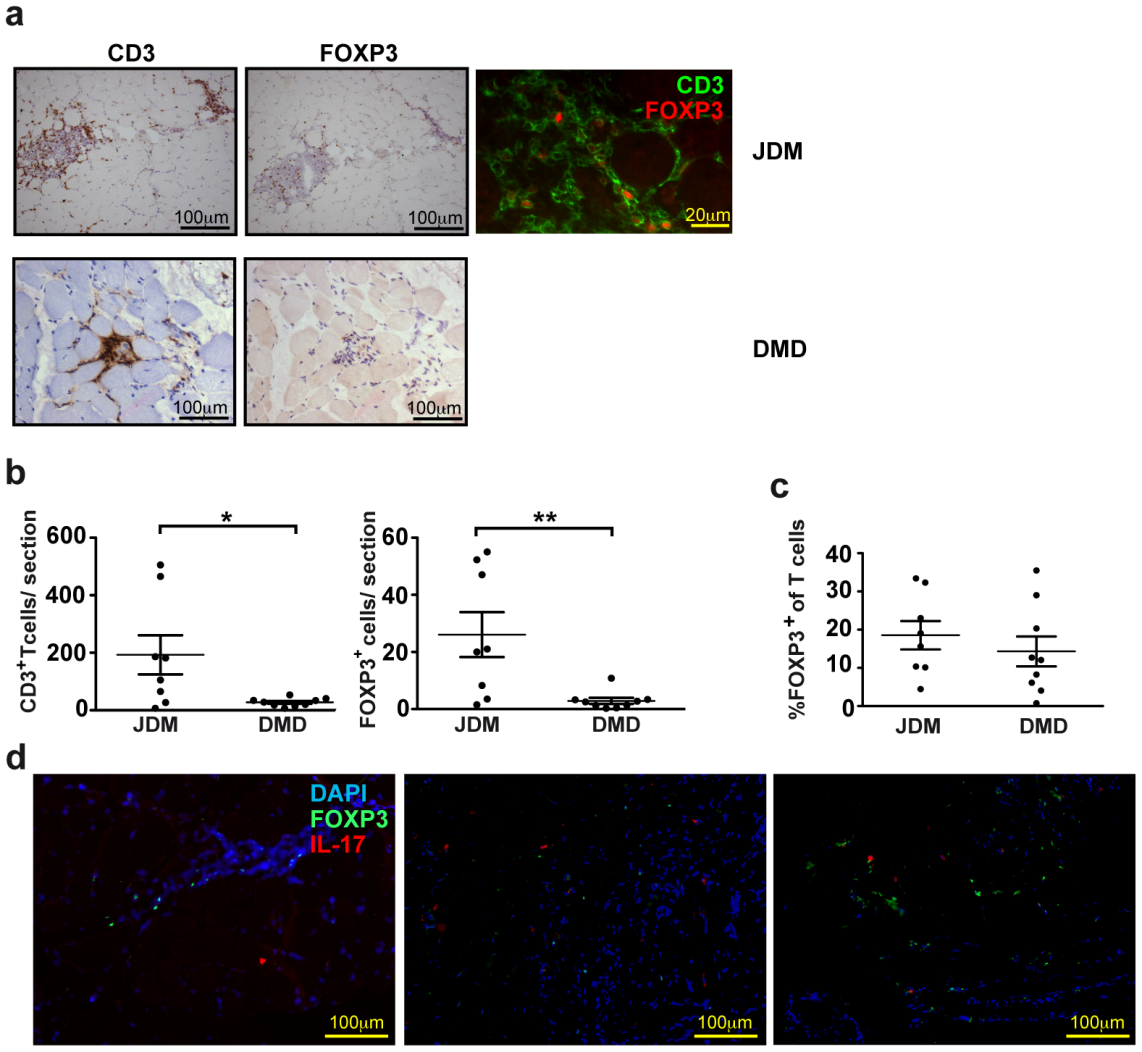
FOXP3^+^ T cells are present in inflamed muscle of newly diagnosed, untreated patients with active JDM. (A) Representative pictures of both immunohistochemistry single stainings for CD3, a marker for T cells (left), FOXP3 (middle) on consecutive sections from muscle biopsies (all at 20× magnification, brown-red indicates positive staining), which were used to analyze cell numbers. The right panel shows an example of immunofluorescence staining (1 out of 4) of a tissue section of a JDM patient, showing FOXP3 (red) expressed in the nuclei of CD3^+^ T cells (green). The upper row shows sections of JDM muscle, the second row shows DMD muscle sections. (B) Average numbers of infiltrated CD3^+^ cells and FOXP3^+^ cells, and (C) percentages of CD3^+^ T cells expressing FOXP3 per section of 8 JDM patients and 9 DMD patients, calculated for each patient, by counting cells from the immunohistochemistry single stainings on consecutive sections, as described in the methods section. Mean ± SEM cell counts: (JDM) CD3 192.7±67.9, FOXP3 26.1±4.8, (DMD) CD3 27.7± 4.8, FOXP3 2.9±1.1, D) Paraffin embedded muscle sections of patients with active JDM were stained with anti-IL-17 (red), FOXP3 (green) and DAPI (blue). Three representative pictures (20× magnification) are shown (N = 3 active JDM patients, at the time of diagnosis, without medication). All graphs show mean percentages ± SEM. *P<0.05, **P<0.01, Mann Whitney U test was used.

Graphs display means ± Standard Error of the Mean (SEM). One-way ANOVA analysis (to compare multiple groups, Figure 1) were used or nonparametric tests Mann Whitney U test (to compare 2 groups, Figure 2). *P<0.05, **P<0.01.

## Results

### JDM patients have normal expression of Treg markers in peripheral blood

In order to establish Treg frequencies in peripheral blood from JDM patients, we analyzed expression of CD25^hi^ according to published methods [9], and FOXP3 by CD4^+^ T cells. For participant characteristics, see Table 1. JDM patients and controls displayed similar percentages of CD25^hi^ (Figure 1A, control: 1.3% ±0.2 SEM, remission: 1.8% ±0.2 SEM, active: 1.8% ±0.2 SEM) and FOXP3 (Figure 1B, control: 7.2% ±0.9 SEM, remission: 6.5% ±0.5 SEM, active: 7.2% ±0.5 SEM) expressing CD4^+^ T cells, independent of disease activity. We observed significantly higher percentages of FOXP3^+^CD4^+^ T cells in JDM patients receiving high corticosteroid treatment (> 1 mg/kg/day, 10.7% ±2.0 SEM), compared to patients receiving low (<0.5 mg/kg/day, 6.1% ±0.5 SEM) corticosteroids (Figure 1C, P<0.05). GITR [21] and CTLA-4 [22] are receptors expressed on Tregs and involved in Treg suppressive function. The expression of Treg receptor GITR was not significantly different between healthy controls and patients (Figure 1D, control: 7.2% ±0.9 SEM, remission: 6.5% ±0.5 SEM, active: 7.2% ±0.5 SEM), while CTLA-4 expression was significantly lower in remitting JDM patients compared to controls, but not significantly different between active and remitting disease (Figure 1E, control: 61.7% ±5.3 SEM, remission: 44.0% ±3.5 SEM, active: 50.6% ±4.0 SEM). Though earlier it was reported that human memory T cells could express both FOXP3 and the inflammatory cytokine IL-17 [16], [17], we could hardly detect any FOXP3^+^IL-17^+^ T cells (Figure 1F). Thus, FOXP3^+^ T cells were present in percentages similar to controls in peripheral blood of JDM patients and did not co-express IL-17.

### FOXP3^+^ T cells are abundantly present in inflamed muscle of patients with active JDM

To investigate whether Tregs are localized at the inflammatory site, we analyzed muscle biopsies from newly diagnosed JDM patients before start of treatment, and compared these to biopsies from Duchenne muscular dystrophy (DMD) patients as non-autoimmune disease controls. Analysis revealed infiltrates of CD3^+^ T cells, of which a proportion expressed FOXP3 (Figure 2A). Numbers of infiltrating T cells (CD3^+^ and CD3^+^FOXP3^+^) per analysed infiltrates muscle area were significantly higher in muscle of JDM patients as compared to DMD (Figure 2B, P <0.05, P<0.01 respectively). Notably, the percentage of FOXP3 within the CD3^+^ T cells, calculated for each patient, did not vary between JDM (18.5% ±3.7 SEM) and DMD (14.3% ±3.9 SEM, Figure 2C). We did not detect any significant correlation with inflammatory or muscle damage markers, or muscle function measurements (CRP, CK, LDH, CMAS) and the % FOXP3 expressing cells in the muscle (Figure S2A). Again, Tregs did not co-express FOXP3 and IL-17 (Figure 2D, showing pictures of 3 separate JDM patients). Thus, compared to peripheral blood (7.2% ±0.5 SEM), high percentages of Tregs (18.5% ±3.7 SEM) were found in inflamed muscle of JDM patients, which seem to be unable to suppress the ongoing inflammation in the muscle.

### Tregs from patients with active JDM exhibit a compromised suppressive function

**Figure 3:**
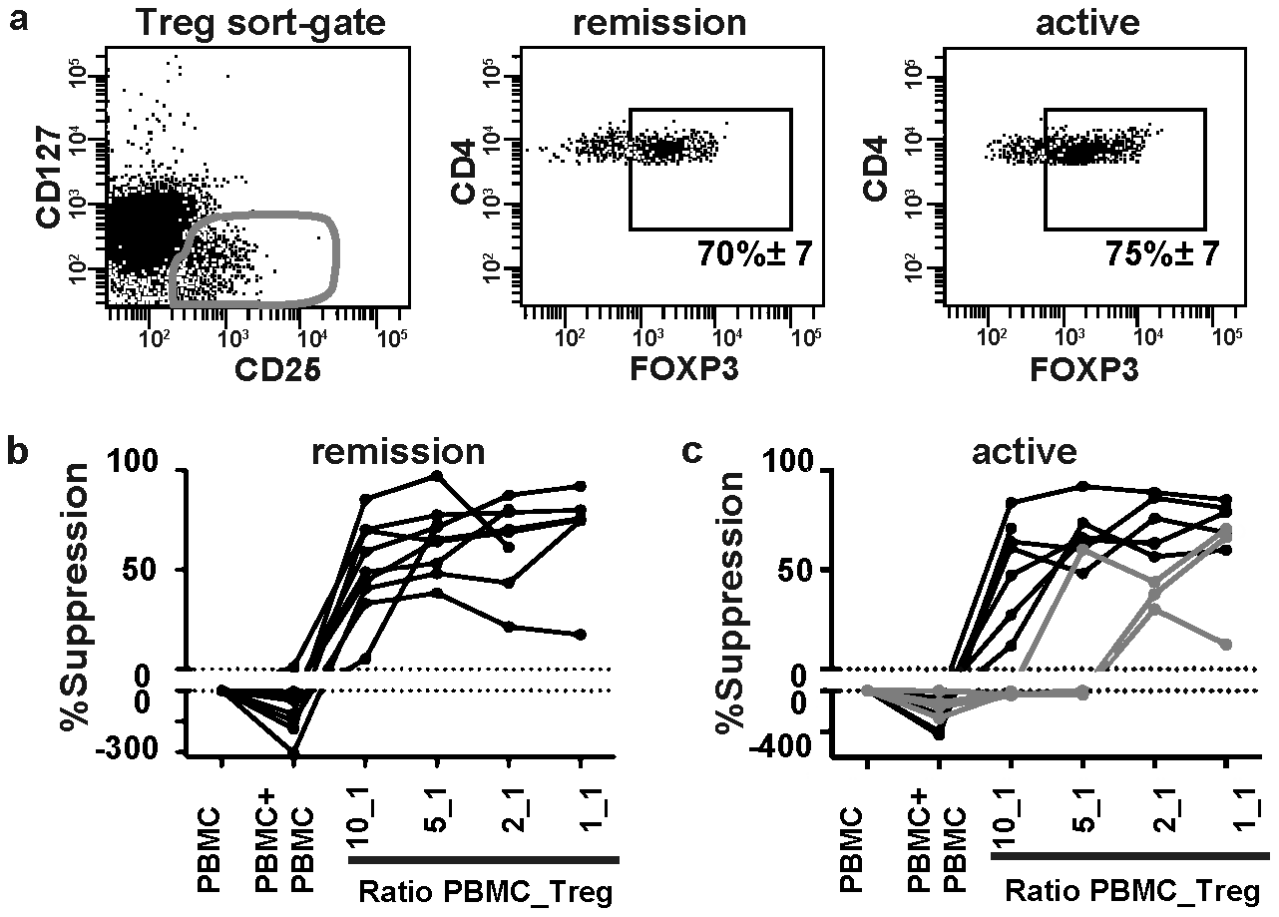
Tregs from patients with active JDM exhibit a compromised suppressive function. (A) Representative dot plots of FOXP3 expression of gated CD4^+^CD25^+^CD127^low^ T cells (left dot plot) of JDM patients in remission (middle dot plot), or with active disease (right dot plot), showing means ± S.E.M. (B, C) CD4^+^CD25^+^CD127^low^ T cells were sorted into co-cultures with anti-CD3 activated autologous PBMC in different ratios. Proliferation was measured on day 6 by ^3^H thymidine incorporation. Level of suppression of PBMC proliferation was calculated by comparison to proliferation of PBMC cultured alone (suppression = 0%). (B) Suppression by CD4^+^CD25^+^CD127^low^ T cells from 9 patients in remission and (C) 11 patients with active disease, grey lines represent 4 patients displaying defective suppression in one or more conditions with Tregs present. Defective suppression is defined as an increased proliferation in the presence of the sorted Tregs, in at least one of the co-culture ratios.

Next, we evaluated the suppressive function of the Tregs. Hereto, CD4^+^CD25^+^CD127^low^ T cells from peripheral blood of JDM patients were sorted. This cell population corresponds at a high percentage to FOXP3 expressing cells, confirming the purity of the sorting (Figure 3A, means ± SEM. remission 70%± 7 SEM, active 75%± 7 SEM). Tregs from patients in remission efficiently suppressed effector T cell activation (Figure 3B). Tregs of 7 patients with active JDM suppressed T cell activation. However, in 4 patients suppression was absent for the cultures containing 10_1, and 5_1 ratios of Tregs (Figure 3C). For [^3^H] thymidine incorporation data see Figure S1 and Table S1. This was neither related to onset or prolonged disease status, nor to use of corticosteroids or other treatments. In addition, we did not detect any significant correlation with inflammatory or muscle damage markers, or muscle function measurements (CRP, CK, ASAT, LDH, CMAS, Figure S2B) though sample size may be too low to determine significance. Furthermore, there were no differences in percentages of Tregs, or PBMC composition (data not shown). Taken together, peripheral blood Tregs from patients with remitting JDM were functionally normal, whereas Tregs from patients with active JDM did not consistently suppress effector T cell responses.

## Discussion

JDM is a complex autoimmune disease with a multifactorial pathogenesis that, among others, involves T cells [6]. In a recently published study we show that chemokines involved in recruitment of inflammatory T cells are elevated in the plasma of patients with active disease onset [3]. Little is known about their pathogenic role, let alone whether Tregs, which are known to be involved in the regulation of autoimmunity, are involved in this process. Here we show the increased presence of Tregs in inflamed muscle of JDM patients, compared to peripheral blood percentages of Tregs. The question is whether these cells could influence the course of JDM. We found no differences in percentages of Tregs between active and remitting JDM in peripheral blood. Interestingly, treatment with high levels (>1 mg/kg) of corticosteroids resulted in increased frequencies of Tregs in active patients. This is in line with previous reports showing that corticosteroid treatment increases Treg numbers in Multiple Sclerosis (MS) patients [23] and in an experimental model for autoimmune encephalomyelitis (EAE) [24]. The expression of Treg receptors CTLA-4 and GITR was not significantly different between patients in remission and patients with active disease. Of note, IL-10 levels in plasma did not show a difference either [3]. The most relevant test available for Treg function is the *in vitro* suppression assay. While Tregs from patients in remission appeared functionally suppressive, Tregs from patients with active JDM did not consistently suppress effector T cells. In 4 out of 11 patient samples, the addition of Tregs did not change effector T cell proliferation, or even resulted in increased proliferation. In both patient groups the level of suppression was variable. Defective suppression was only seen in the lower ratios of Tregs (10_1 and 5_1), which are more physiologically relevant, since the higher ratios probably overrule any defects. Defective suppression was not related to treatment with corticosteroids, although patient numbers may be too small to establish a significant effect. A defect in suppressive capacity could be due to the pro-inflammatory environment in active disease affecting the function of Tregs in the periphery. In addition, functional Tregs may have migrated into the inflamed tissue, in the case of JDM patients to muscle and skin. However, since JDM is not a self-limiting disease, it is certain that the Tregs present in the muscle of active JDM patients are not sufficient to control muscle inflammation. These findings are consistent with reports in JIA patients; JIA synovial fluid contains high frequencies of FOXP3^+^ Tregs, which cannot prevent inflammation [9], [25]. This may be attributed to a defect in Treg function or a transient upregulation of FOXP3 in non-suppressive T cells. The proportions of FOXP3^+^ T cells are similar in JDM and DMD muscle, suggesting that Tregs are able to infiltrate the inflamed muscle independent of the underlying cause of inflammation. We do not see T cell infiltrates in healthy muscle sections (not shown). A recent study in mouse models of muscle injury and DMD, showed that Tregs are present in high proportions in the muscle upon injury and inflammation, while in controls there were very low numbers of infiltrated cells [26]. Altogether, these mouse models confirm our finding that high proportions of FOXP3^+^ T cells accumulate in muscle upon inflammation.

Under inflammatory conditions human FOXP3^+^ Tregs co-express pro-inflammatory cytokines such as IL-17 [16], [17]. Even though IL-17 is implicated in immune pathogenesis of myositis in adults [27], [28], Tregs from JDM patients did not co-express IL-17 in blood or muscle. Of note, IL-17 is not elevated in plasma of JDM patients either [3]. An alternative explanation for the impaired suppression observed in active JDM patients, may be resistance of effector T cells towards Treg-mediated suppression: Synovial fluid effector T cells from JIA patients could not be suppressed by Tregs, while the synovial Tregs could suppress peripheral blood effector T cells [29], [30]. In the future, such cross-over suppression assays should establish whether effector T cells are resistant to suppression in JDM. The clinical course of JDM can be either monophasic cyclic or chronic [31],and so far little is known concerning the immune regulatory effects that may influence the outcome. Here we have shown that Tregs may be involved in the regulation of JDM inflammation. Future prospective studies should determine whether Tregs are able to actively influence the differential disease course in JDM, and more importantly, whether manipulation of Tregs and/or resistant effector T cells could be a therapeutic option in JDM.

## Supporting Information

Figure S1 **Raw proliferation data of the suppression assays for each patient.** (A) Proliferation of PBMC cultured with or without Tregs from 9 patients in remission and (B) 11 patients with active disease. Grey lines represent 4 patients displaying defective suppression (increased proliferation in at least one of the Treg ratios). Depicted are [3H] thymidine cpm (counts per minute). Shown are mean ± SEM of triplo or duplo measurements per co-culture condition.
(PDF)

Figure S2 **Correlation analyses of disease course markers and Treg percentages and function.** (A) Correlation analyses of disease course markers and Treg percentages in muscle of JDM patients at active onset. (B) Correlation analysis of disease course markers and peripheral blood Treg suppressive function in JDM patients. Analysis was performed for each coculture ratio of PBMC:Tregs (10∶1, 5∶1, 2∶1, 1∶1).
(PDF)

Table S1 **Counts per minute (cpm) of ^3^H thymidine incorporation of all suppression assays.** Depicted are all raw values of ^3^H thymidine cpm for each single well analyzed in the suppression assays. Labeled in blue are the assays displaying defective suppression in 1 or more conditions with Tregs. Labeled in grey are the assays we excluded from the analysis in the graphs, since PBMC+PBMC shows lower proliferation than PBMC alone, suggesting that merely an increase of cell numbers lowered the proliferation of the cells.
(PDF)
