# Supplementary material for: Increased Presence of FOXP3+ Regulatory T Cells in Inflamed Muscle of Patients with Active Juvenile Dermatomyositis Compared to Peripheral Blood
Source: PLoS One. 2014 Aug 26;9(8):e105353. doi: 10.1371/journal.pone.0105353 (PMC4144849; doi:10.1371/journal.pone.0105353)
Supplement: Figure S1 — Raw proliferation data of the suppression assays for each patient. (A) Proliferation of PBMC cultured with or without Tregs from 9 patients in remission and (B) 11 patients with active disease. Grey lines represent 4 patients displaying defective suppression (increased proliferation in at least one of the Treg ratios). Depicted are [3H] thymidine cpm (counts per minute). Shown are mean ± SEM of triplo or duplo measurements per co-culture condition. (PDF) [file pone.0105353.s001.pdf]

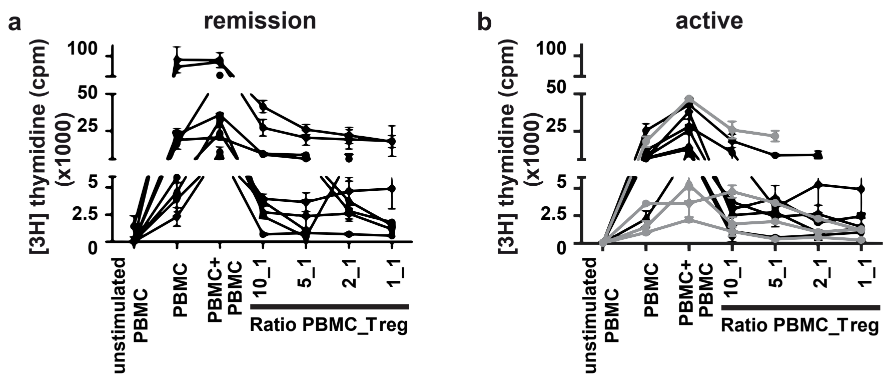

Figure S1. Raw proliferation data of the suppression assays for each patient.
(A) Proliferation of PBMC cultured with or without Tregs from 9 patients in remission and (B) 11 patients with active disease. Grey lines represent 4 patients displaying defective suppression (increased proliferation in at least one of the Treg ratios). Depicted are [3H] thymidine cpm (counts per minute). Shown are mean ± SEM of triplo or duplo measurements per co-culture condition.
