# Supplementary material for: Increased Presence of FOXP3+ Regulatory T Cells in Inflamed Muscle of Patients with Active Juvenile Dermatomyositis Compared to Peripheral Blood
Source: PLoS One. 2014 Aug 26;9(8):e105353. doi: 10.1371/journal.pone.0105353 (PMC4144849; doi:10.1371/journal.pone.0105353)

Supplementary Figure S2

A)Correlation analyses of disease course markers and Treg percentages in muscle of JDM patients at active onset.

Correlations

			FoxP3	CRP	CK	LDH	CMAS
Spearman's rho	FoxP3	Correlation Coefficient	1.000	.211	-.359	-.707	.211
		Sig. (2-tailed)		.616	.382	.050	.616
		N	8	8	8	8	8
	CRP	Correlation Coefficient	.211	1.000	-,719*	-.575	-.030
		Sig. (2-tailed)	.616		.045	.136	.944
		N	8	8	8	8	8
	CK	Correlation Coefficient	-.359	-,719*	1.000	,738*	-.323
		Sig. (2-tailed)	.382	.045		.037	.435
		N	8	8	8	8	8
	LDH	Correlation Coefficient	-.707	-.575	,738*	1.000	-.204
		Sig. (2-tailed)	.050	.136	.037		.629
		N	8	8	8	8	8
	CMAS	Correlation Coefficient	.211	-.030	-.323	-.204	1.000
		Sig. (2-tailed)	.616	.944	.435	.629	
		N	8	8	8	8	8

*. Correlation is significant at the 0.05 level (2-tailed).

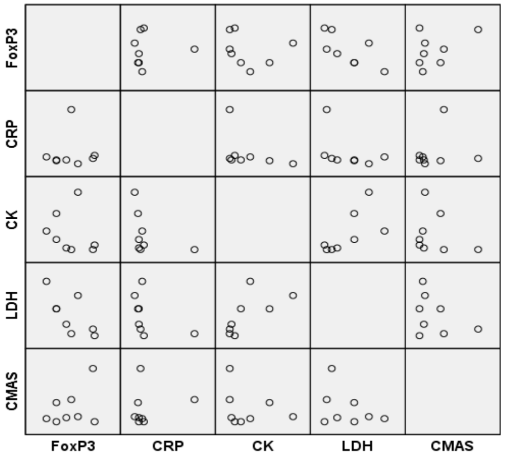

B) Correlation analysis of disease course markers and peripheral blood Treg suppressive function in JDM patients.

Analysis was performed for each coculture ratio of PBMC:Tregs (10:1, 5:1, 2:1, 1:1).

Correlations

		supp 10:1	supp 5:1	supp 2:1	supp 1:1
CRP	Correlation Coefficient	-.347	-.276	-.495	-.655
	Sig. (2-tailed)	.296	.440	.175	.078
	N	11	10	9	8
CK	Correlation Coefficient	.073	-.394	-.467	-.381
	Sig. (2-tailed)	.832	.260	.205	.352
	N	11	10	9	8
ASAT	Correlation Coefficient	-.155	-.442	-.283	-.405
	Sig. (2-tailed)	.650	.200	.460	.320
	N	11	10	9	8
LDH	Correlation Coefficient	-.064	-.552	-.383	-.476
	Sig. (2-tailed)	.853	.098	.308	.233
	N	11	10	9	8
CMAS	Correlation Coefficient	-.388	-.555	-.521	-.431
	Sig. (2-tailed)	.238	.096	.150	.286
	N	11	10	9	8

no significant correlation

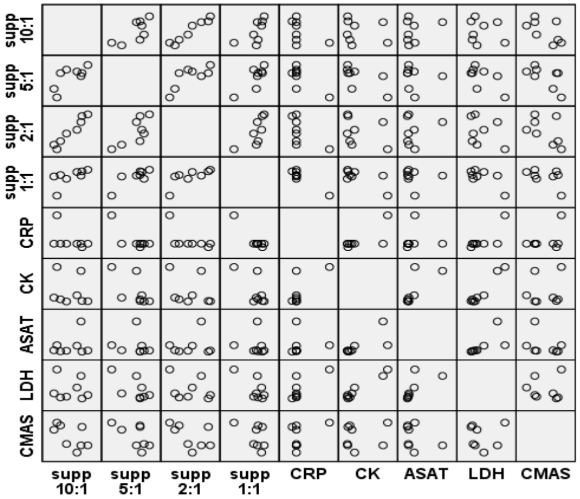

Supplement: Figure S2 — Correlation analyses of disease course markers and Treg percentages and function. (A) Correlation analyses of disease course markers and Treg percentages in muscle of JDM patients at active onset. (B) Correlation analysis of disease course markers and peripheral blood Treg suppressive function in JDM patients. Analysis was performed for each coculture ratio of PBMC:Tregs (10∶1, 5∶1, 2∶1, 1∶1). (PDF) [file pone.0105353.s002.pdf]
