# Supplementary material for: Increased Presence of FOXP3+ Regulatory T Cells in Inflamed Muscle of Patients with Active Juvenile Dermatomyositis Compared to Peripheral Blood
Source: PLoS One. 2014 Aug 26;9(8):e105353. doi: 10.1371/journal.pone.0105353 (PMC4144849; doi:10.1371/journal.pone.0105353)
Supplement: Table S1 — Counts per minute (cpm) of 3H thymidine incorporation of all suppression assays. Depicted are all raw values of 3H thymidine cpm for each single well analyzed in the suppression assays. Labeled in blue are the assays displaying defective suppression in 1 or more conditions with Tregs. Labeled in grey are the assays we excluded from the analysis in the graphs, since PBMC+PBMC shows lower proliferation than PBMC alone, suggesting that merely an increase of cell numbers lowered the proliferation of the cells. (PDF) [file pone.0105353.s003.pdf]

Vercoulen et al., Supplementary Table S1

cpm (3H counts, raw data)

defective suppression in 1 or more conditions with Tregs

excluded from graphs: PBMC+PBMC shows lower proliferation than PBMC alone.

Remission

	Patient#	1			2			3			4			5			6		
PBMC 10,000 without aCD3	PBMC no aCD3	505.5	109.4		10	22	81	39	114	90	384	565	3369	19	30	40	93	60	
PBMC 10,000	PBMC	4704.9	4234.7		1656	24100	22862	26887	121048	125889	81316	60777	80454	2517	3633	735	5958	896	4925
PBMC 20,000	PBMC+PBMC	2675.9	3730.9		55622	56158	47470	126757	64720	79149	101729	75171	81250	17982	7814	6805	7956	7524	7708
10,000PBMC+ 1,000 Tregs	10_1	2148.3	1457		2384	2338		34084	16024	31581	41733	33917	48653	1193	699	187	3359	4088	
10,000PBMC+ 2,000 Tregs	5_1	516.5	814.1		567	357		14550	16398	30675	20956	32870	24199	1341	760	365	991	1151	
10,000PBMC+ 5,000 Tregs	2_1	1891.9	1041.1		9354	3207		32461	11296	14452	12358	31709	22139	1177	712	259			
10,000PBMC+ 10,000 Tregs	1_1	1307.3	797.6					4460	38630	11577	14887	26108	12805	793	466	457			

	Patient#	7			8			9			10			11			12		
PBMC 10,000 without aCD3	PBMC no aCD3	56	82		93	97		76	27	35	48	43	31	43	38	39	18	25	34
PBMC 10,000	PBMC	25251	12841		11436	11725		2761	6408		22596	33676		5807	5381	6608	27484	25856	14247
PBMC 20,000	PBMC+PBMC	18745	22476		5226	7497		27590	37190		21874	26665		15536	26636	28530	39658	32756	35475
10,000PBMC+ 1,000 Tregs	10_1	9560	9904		6515	7315		2163	3293		17630	5998		4824	3609	3499	11128	8833	7664
10,000PBMC+ 2,000 Tregs	5_1	8640	9125		3195	4559		869	3890		2454	7820		5120	3762	2098	6936	7867	4536
10,000PBMC+ 5,000 Tregs	2_1	1894	5595					2102	3098		1808	1849		5526	6353	2120	3238	3071	2207
10,000PBMC+ 10,000 Tregs	1_1							1206	1111		2243	1748		7927	5294	1485	1958	1818	1659

Active JDM

	Patient#	1			2			3			4			5			6		
PBMC 10,000 without aCD3	PBMC no aCD3	50	51		85	17	17	71	126	38	34	45	48	27	38	36	14	33	23
PBMC 10,000	PBMC	23246	11381	19743	3258	2615	754	29283	16361	30743	17458	1895	4225	4433	8187	8812	1070	2574	733
PBMC 20,000	PBMC+PBMC	42674	50702	47093	13926	8069		38351	38108	51944	11191	6867	58964	14457	15402	13832	7281	6236	2436
10,000PBMC+ 1,000 Tregs	10_1	31599	20259		198	73	1660	14041	22979		2109	1224	5923	3775	3781		2485	2269	521
10,000PBMC+ 2,000 Tregs	5_1	18115	25423					8326	9546		1954	6077	4207	2649	2182		2493	1414	2029
10,000PBMC+ 5,000 Tregs	2_1							6469	12018		2070	1719		1847	3456		745	1149	1163
10,000PBMC+ 10,000 Tregs	1_1										2778	2135		227	2780		1357	1023	1452

	Patient#	7			8			9			10			11			12			13		
PBMC 10,000 without aCD3	PBMC no aCD3	50	36		46	29		28	20		48	31	32	27	34	25	29	18	32	29	59	40
PBMC 10,000	PBMC	3722	3488		1678	2254		1451	2151		8378	15936	12484	2964	13249	3827	1192	678	1079	12663	5928	2764
PBMC 20,000	PBMC+PBMC	2583	6160	2160	1677	1458	816	801	1720		35862	29734	19044	8533	14306	16431	2108	1856	2462	51005	21689	41359
10,000PBMC+ 1,000 Tregs	10_1	4145	5266		1622	1490		920	2233		24604	6249	1651	1552	786	954	383	1035	1788	4208	1713	1752
10,000PBMC+ 2,000 Tregs	5_1	3410	3926		1100	2113		743	1909		2739	2185	4815	366	822	443	527	104	549	3135	4974	377
10,000PBMC+ 5,000 Tregs	2_1	2317	2177		992	1673		557	660		10492	2097	3394	187	385	1647	400	383	875	915	1358	704
10,000PBMC+ 10,000 Tregs	1_1	1373	1045					486	265		9512	3001	2232	626	1498	852	268	172	429	509	3208	361

Average 3H counts

Remission

	Patient#	1	2	3	4	5	6	7	8	9	10	11	12
PBMC 10,000 without aCD3	PBMC no aCD3	307.45	37.6666667	81	1439.33333	29.6666667	76.5	69	95	46	40.6666667	40	25.6666667
PBMC 10,000	PBMC	4469.8	16206	91274.6667	74182.3333	2295	3926.333333	19046	11580.5	4584.5	28136	5932	22529
PBMC 20,000	PBMC+PBMC	3203.4	53083.3333	90208.6667	86050	10867	7729.333333	20610.5	6361.5	32390	24269.5	23567.3333	35963
10,000PBMC+ 1,000 Tregs	10_1	1802.65	2361	27229.6667	41434.3333	693	3723.5	9732	6915	2728	11814	3977.33333	9208.33333
10,000PBMC+ 2,000 Tregs	5_1	665.3	462	20541	26008.3333	822	1071	8882.5	3877	2379.5	5137	3660	6446.33333
10,000PBMC+ 5,000 Tregs	2_1	1466.5	6280.5	19403	22068.6667	716		3744.5		2600	1828.5	4666.33333	2838.66667
10,000PBMC+ 10,000 Tregs	1_1	1052.45		18222.3333	17933.3333	572				1158.5	1995.5	4902	1811.66667

Active JDM

	Patient#	1	2	3	4	5	6	7	8	9	10	11	12	13
PBMC 10,000 without aCD3	PBMC no aCD3	50.5	39.6666667	78.3333333	42.3333333	33.6666667	23.33333333	43	37.5	24	37	28.6666667	26.3333333	42.6666667
PBMC 10,000	PBMC	18123.3333	2209	25462.3333	7859.33333	7144	1459	3605	1966	1801	12266	6680	983	7118.33333
PBMC 20,000	PBMC+PBMC	46823	10997.5	42801	25674	14563.6667	5317.666667	3634.33333	1317	1260.5	28213.3333	13090	2142	38017.6667
10,000PBMC+ 1,000 Tregs	10_1	25929	643.666667	18510	3085.33333	3778	1758.333333	4705.5	1556	1576.5	10834.6667	1097.33333	1068.66667	2557.66667
10,000PBMC+ 2,000 Tregs	5_1	21769		8936	4079.33333	2415.5	1978.666667	3668	1606.5	1326	3246.33333	543.666667	393.333333	2828.66667
10,000PBMC+ 5,000 Tregs	2_1			9243.5	1894.5	2651.5	1019	2247	1332.5	608.5	5327.66667	739.666667	552.666667	992.333333
10,000PBMC+ 10,000 Tregs	1_1				2456.5	1503.5	1277.333333	1209		375.5	4915	992	289.666667	1359.33333

% suppression

Remission

	Patient#	1	2	3	4	5	6	7	8	9	10	11	12
PBMC 10,000	PBMC	0	0	0	0	0	0	0	0	0	0	0	0
PBMC 20,000	PBMC+PBMC	28.3323639	-227.553581	1.16790347	-15.997969	-373.507625	-96.8588165	-8.21432322	45.0671387	-606.51107	13.7421808	-297.291526	-59.6298105
10,000PBMC+ 1,000 Tregs	10_1	59.6704551	85.4313217	70.1673338	44.1452817	69.8039216	5.165973342	48.9026567	40.2875524	40.4951467	58.011089	32.951225	59.1267551
10,000PBMC+ 2,000 Tregs	5_1	85.1156651	97.149204	77.4953985	64.9399902	64.1830065	72.72264199	53.3629108	66.5213074	48.0968481	81.7422519	38.3007417	71.3865092
10,000PBMC+ 5,000 Tregs	2_1	67.1909258	61.2458349	78.7421848	70.2507785	68.8017429		80.3397039		43.2871633	93.5012084	21.3362553	87.3999438
10,000PBMC+ 10,000 Tregs	1_1	76.4542038		80.0357164	75.8253313	75.0762527				74.7300687	92.9076628	17.3634525	91.9585127

Active JDM

	Patient#	1	2	3	4	5	6	7	8	9	10	11	12	13
PBMC 10,000	PBMC	0	0	0	0	0	0	0	0	0	0	0	0	0
PBMC 20,000	PBMC+PBMC	-158.35755	-397.849706	-68.0953565	-226.668929	-103.858716	-264.473384	-0.8136847	33.0111902	30.0111049	-130.012501	-95.9580838	-117.904374	-434.081011
10,000PBMC+ 1,000 Tregs	10_1	-43.0697076	70.8616267	27.3043843	60.7430656	47.1164614	-20.5163354	-30.5270458	20.854527	12.4652971	11.6691125	83.5728543	-8.71481858	64.0693046
10,000PBMC+ 2,000 Tregs	5_1	-20.1158727		64.9050231	48.0956824	66.1884099	-35.6180032	-1.74757282	18.2858596	26.3742365	73.5338877	91.8612774	59.9864361	60.2622337
10,000PBMC+ 5,000 Tregs	2_1			63.6973569	75.894902	62.8849384	30.15764222	37.6699029	32.2227874	66.2132149	56.5655742	88.9271457	43.7775517	86.0594708
10,000PBMC+ 10,000 Tregs	1_1				68.7441683	78.9543673	12.45145077	66.4632455		79.150472	59.9298875	85.1497006	70.5323839	80.9037696
